# Indents

**Published:** 1906-06-15

**Authors:** 


### INDENTS.

☞ **Brief Articles of Technical or Scientific Value will receive notice and comment. Contributions invited.**

**Death from Nitrous Oxid.** A man, forty-four years of age took the gas in the office of Dr. Moork, in New York City and died while under its influence. The man's physician said he could safely take the gas.

**Restrict the Flow of Solder.** In filling the cusps of gold crowns mark the limits of the solder on the sides of the crown with a lead pencil and the solder will not flow over the pencil mark.—F. W. Franklin, *Western Journal.*

**Selecting a Shade for Crown.** Place the crown in place and hold a large mouth mirror back of it and compare the colors in the mirror. The shades will appear more sharply in contrast when viewed in this way than by direct view.—P. P. Dorr, in *Brief.*

**Resin-Soap the Belt.** Dr. W. W. Belcher says the grip of the engine cord or belt is greatly improved by holding against it while running at high speed a bar of soap containing resin. To make sure of the resin, buy cheap laundry soap.

**The Gold Matrix: Protection from Fusing.** It is not necessary that any matrix be invested; when gold is used it is protected from fusing by coating it, preferably with rouge because of its great fineness and affinity for a smooth surface. The rouge is bought in powder and spatulated with alcohol and water. It will resist heat to an astonishing degree.—W. A. Capon, *Items of Interest.*

**Inlaying a Gold Filling.** Burnish a piece of heavy No. 30 or 60 gold foil into the cavity and cement it to place. Then fill the cavity with gold in the usual way. This insures marginal contact and adhesion, consequently a water-tight filling.—*British Journal.*

**To Make Hard Plaster Models.** Mix the plaster of Paris with one-sixth of its volume of pulverized slaked lime. After the model is dried place it in a 10 percent solution of zinc sulphate until it is thoroughly saturated.—Remove and dry.—*Dental Era.*

**A Printer's Blunder.** A Western society editor wrote that a certain young lady's breast was filled with rage, but the printer got it rags. That editor is now said to be camping on a near-by hill crest from which he can have an unobstructed view in all directions for several miles.

**Bake, not Broil, Porcelains.** Dr. J. E. Nyman says: "I am quite confident that we have all been fusing our porcelains too quickly. You can obtain the same result by prolonging the time of the exposure, and cutting down the intensity. We have been broiling our porcelain instead of 'baking' it."

**Decoloring Stained Instruments.** The discoloration or coating which results from the frequent sterilization of instruments by boiling, may be removed by rubbing them with a cloth saturated with a aqueous solution of two ounces each of prepared chalk, ammonia and alcohol.—Byram, in *Review.*

**To Prevent Rust.** Paraffin oil is the best preservative against rust. The most convenient way of applying it is to dissolve one part of the oil in 200 parts of benzine, plunging the instruments into the solution after being thoroughly dried and warm. Allow the benzine to evaporate in a dry room.—*Medical Standard.*

**Keep Broaches from Rusting.** Sterilize as usual and clean by pushing them through the steel wire brush wheel instead of pulling. Then dip them in the oil called "Three in One," and put away for use.—R. B. Calvin, in *Brief*.

We keep our broaches in small bottles with cotton in the Bottom, saturated with glymol and lysol.—Ed. REGISTER

**Retention of Dentures.** If possible two roots should be left in each jaw and utilized to support the plate. A satisfactory method is to fit each such root with a gold cap and tube, into which fit a pin attached to the plate. The stability which even one root so treated will give to an entire denture is surprising.—Wm. M. Gabriel, *Dental Record*.

**Alypin.** This is a new local anesthetic which it is hoped may take the place of cocain. It anesthetizes the mucous membrane when rubbed on it, and injecting it produces similar effects. So far as it has been investigated it seems to have no advantages over cocain as its toxic effects are about the same, besides causing undesirable heart complications.—March *Cosmos*.

**To Prevent Adhesion of Cement under Bridges.** To avoid the danger of getting cement under the saddle of a bridge, cover all portions where it is not desired to have the cement stick, with gum acacia dissolved in water. If cement goes where it is not wanted, the gum will dissolve from underneath and the cement will not stick.—Dr. Geo. W. Whitfield, in *Dental Review*.

**Protective for Porcelain during Soldering.** When circumstances make it necessary to near-by soldering to invest crowns or bridges porcelain-face up, cover the porcelain with thin asbestos paper saturated with the investment mixture, catching the free ends in the body of the investment proper. The paper protects throughout the operation from the direct action of the flame.

**Root Treatment.** "Next time you want to get a liquid medicament to the end of a root canal do it this way: Flood the canals with the liquid, introduce a Downie broach and revolve it the wrong way; the blades of the broach will operate like the screw propeller on a steamboat. Let me know what you think of the idea."

Its "all right."—*Office and Laboratory.*

**Spence Metal.** Spence metal is composed of 60 parts of sulphite of iron and 40 parts of sulphur. The fusing point is 225 degrees Fahrenheit. It is of great value when used as an articulating model, by minimizing the attrition of the antagonistic surfaces, which results when teeth or porcelain are brought into repeated contact with teeth of a plaster model.—*International Dental Journal.*

**Antiseptic and Anesthetic Paste.**

| | | |
|---|---|---|
| ℞ | White Vaseline | ℥ j |
| | Cocain | grs. xiv |
| | Menthol | grs. xxiv |
| | Oil of Peppermint | grs x |
| | Chloretone | grs. jx |
| | Phenol | grs. ij |

Mix and apply before scaling the teeth by rubbing it into the spaces between the teeth and on the gums.

**Office Coats.** The "crying need" of the dentist who would be decently dressed in his office is an office coat. This should be of linen, duck, or similar material; washable, and plain even to the absence of pockets. Those now in general use, decorated as many of them are with four pockets, a belt and four sets or frogs make a wearer look like a ring master just escaped from a dog circus. A coat without ginger-bread should meet with a large scale.

**To Anchor Plastic Fillings.** Double headed pins or rivets made from No. 23 brass wire, which has been gold plated, cut to assorted lengths, furnish a secure anchorage for large fillings in badly decayed teeth. They can be cemented into suitable places at contra angles so as to furnish good anchorages. Platinum pins from old teeth may be similarly used, also small screws used by cabinet makers.—W. S. Payson, in *Items*.

**Repairing Spots in Pink Rubber.** When the dark colored vulcanite shows through the pink dentures made of that material a remedy is to cut out the dark spot to a sufficient depth and make an inlay of pink, either out of an old denture or from a piece vulcanized especially and kept on hand for these accidents. This same plan is useful where a small piece of plaster has made an ugly looking pit in any portion of the denture.—C. W. Davidson, in *Western Journal*.

**Dish for Acid Bottles.** Set your laboratory acid bottles in a glass dish, or granite or other glazed tray, first covering the bottom with a thin layer of washing soda. The last mentioned article takes up the drip from the neck of the bottles, also to some degree the heavy fumes. If it is desired to have a dish to each bottle, the sponge dishes in use by bank cashiers and dentists generally when counting their money, answers the purpose perfectly.—*Office and Laboratory*.

**Dental Styptic.** To check hemorrhage from a root socket roll pledgets of bibulous paper as solid as possible slightly smaller than the opening of the socket. Soak a ball in pure creosote, squeeze out excess and pack it into the socket as far as possible. Make other similar balls and pack the socket until it will contain no more. If the hemorrhage has been extreme apply a compress on top of the balls, but in most cases the compass will not be needed.—J. E., in *Brief*.

**Proximal Cavities.** When, in excavating a compound cavity in a tooth, you find a spot upon the next tooth, opposite the cavity, if the patient is young and the caries active, that should be cut out at once and the cavity filled. On the other hand, if the patient is comparatively old and one in whose mouth the progress of caries is stopping, I believe you are not justified in cutting it out. There is a difference in the conditions which must be taken into consideration.—F. B. Noyes, in *Review.*

**Collect Your Bills.** Persons who owe bills which are past due, if in need of further dental services will seldom go to the dentist whom they owe to have it done, even when they expect to pay for that particular work. But when they are forced to pay the old bill they are as likely to return for further work to the original dentist, as to go elsewhere; more likely, if the work has been satistactory, and work which has been paid for is usually the most satisfactory.—Dr. H. G. Logan, in *Dental Review.*

**Setting a Davis Crown Pin.** Prepare the crown and pin in the usual manner; fill the socket in the crown with powdered sulphur; pass the crown through an alcohol flame until the powders liquifies; insert the pin and hold it in place until cool.

Pins fastened into crowns in the above manner cannot be removed without breaking the crown. This method is good in setting pinless teeth in bridge-work on all removable tooth bridges.—J. B. Merrick, in *Western Journal.*

**Woman's Dental Association.** The annual meeting of the Women's Dental Association was held at 1716 Chestnut Street, Philadelphia, March 3, 1906. Dr. H. Belle Whitcomb presided. The following officers were elected tor the year 1906-1907: Emily W. Wyeth, president; H. Belle Whitcomb, vice-president; Rebecca R. Cornish, corresponding secretary; Eliza Yerkes, recording secretary; Elizabeth A. Davis McDonald, treasurer. Executive Committee:—Frances Crouch, Annie L. Locht, Martha Corkhill, Anna K. Leaming, Matilda Grath.—Eliza Yerkes, Secretary, Philadelphia, Pa.—*Brief.*

**Preventing Caries.** As soon as possible after the teeth back of the cuspids have erupted, dry them carefully and swab them with a solution of nitrate of silver, working it into the sulci and any defective structure that would invite caries. After a minute or so wipe off the teeth. I have used this method of preventing caries with success for the past ten years. It will be necessary to watch the teeth carefully and should any indication of caries appear repeat the treatment.—H. L. Hamilton, in *International.*

**Too Much Rubber.** With a good model and good under cuts it frequently happens that the plate does not stick. In ninety percent of the cases it is because too much pressure is used in closing the flask, making irregular compression of the model. To prevent this use the cloth (wet) that comes on the sheets of rubber, and open the flask two or three times, removing excess from waste gates. The last time when the flask has come together remove the cloth. Try this plan and see results.—G. G. Brown, in *Western Journal.*

**Dental Caries and Osteoclasts.** Drs. Coyne and Cuvalie, of the Pathological Laboratory of Bordeaux, in their examination of microscopical specimens of carious teeth, think they have detected in the zones of decalcifying dentine multinuclar cells resembling osteoclasts. They divide the affected region into four layers. The zone of destruction, the zone of decalcification, the opaque zone and the transparent zone. In the first two they claim to have plainly detected the osteoclasts, which they believe participate in the tooth disintegration. March *Cosmos.*

**Correct Bite.** To get a properly related bite of an edentulous mouth, make the base plate as usual and add the wax for contour and occlusal effects. At the back part of the upper trail plate and on the lingual surface build on a clutch, or semi-circular ring of wax that will admit the end of the tongue. Place the bite plates in the mouth and tell the patient to close his lips and place his tongue in the clutch on the upper plate and then bring his jaws together, biting on the back teeth. The position of the tongue will center the occlusion and the closure of the lips prevent protruding the lower jaw.—R. Kessel, in *Items.*

**Sterilizing the Mouth.** Dr. H. C. Register, in the May *Brief*, says that he has a number of patients who visit him every ten days for treatment of the mouth. He uses a germicidal wash that is colored, so that it will stain any mucoid deposits on the teeth. He uses it as a spray with high pressure of condensed air and blows it in between the teeth and into alveolar pockets. If any of the teeth are not cleaned by it the coloring material will indicate the places where hand polishing with abrasives should be used to completely free the teeth from any deposits that is likely to set up caries, or to serve as a focus of inflection.

**Richmond Metal.** This metal offers the great advantages of very low fusing point—160 degrees, fifty-two below boiling water, enabling you to run a model over a gutta-percha impression. The formula is as follows: Tin, 20 parts by weight; lead, 19 parts by weight; cadium, 13 parts by weight; bismuth, 48 parts by weight. In swagin a matrix take impression of cavity with gutta-percha, invest in plaster. It is not necessary to wait for plaster to thoroughly harden or dry; two minutes is ample time when you can run the Richmond metal giving a metal die on which to swage a matrix.—I. P. Root, *Kansas City Dental Journal.*

**Preventing Dental Caries.** The best time to stop a cavity is before it forms. It has been said, "Micro-organisms attach themselves to the tooth surfaces," and that a further step is a slight roughening of the enamel surface, which roughening should all be cut away. If this slightly roughened surface be polished perfectly and *kept polished,*

further steps of decay are impossible, hence cutting away is needless. Perfect polishing of the teeth and constant maintenance of the polish will make needless this vast waste of tooth structure in so-called vulnerable positions and even *prevent altogether* the formation of cavities.—L. H. Arnold, in *Review*.

**Investing Bridge-work.** The investment should be trimmed down until no surplus beyond the actual requirements remain, and until all the wax is exposed. This in turn facilitates the free exposure of the metal parts, and diminishes the degree of heat required to solder.

The wax should be carefully removed with a small, sharp pointed instrument, and any overhanging edges of the investment then trimmed away until the metal surfaces of both "abutment pieces" and dummies are freely exposed. Strength in the investment should be obtained from the under side in order to admit of this, and thus facilitate and expedite the process of heating up and soldering.—Hart J. Goslee, in *Items of Interest*.

**Value of Dentistry.** Without the aid of skillful dentistry, humanity could not have health and attain longevity. We are, therefore, as practitioners of a branch of the healing art, important factors, and cannot be dispensed with. My humble opinion is that in the sphere we operate, we are quite as necessary as is the physician and surgeon, for it takes the combined skill of the three professions to keep the human body in a fair state of health. Our remedy then lies in the appreciation of this fact, and a fee commensurate with service rendered should be considered in every case we treat, every case we operate upon and every denture we supply.—W. H. Chilson, in *Review*.

**Handling Sore Teeth.** For this purpose some tie a string to the tooth, instructing the patient to pull to the degree of tooth comfort. Other sesort to modelling compound moulded about the sore teeth and its pain-free neighbors. Either means answers the purpose, but for speed, comfort and convenience, we use the orangewood wedgee or a pair of them, as in the ordinary wedging operation preparatory to filling. The sore tooth is rendered incapable of yielding a symptom of pain. This method of fixing teeth applies also in cases where soreness is absent; it kills the vibration attending the use of burs, and especially those eunvenly or too coarsely cut.—*Office and Laboratory.*

**Treatment of Pyorrhea.** First inject into the pockets a few drops of a 15 percent solution of cocain until the tissues are anesthetized; then with a set of Young's pyorrhea instruments, as revised by Dr. Good, I go up, if necessary, to the end of the roots, being careful to remove perfectly all of the calcareous deposits at the first sitting, and when I am satisfied there is nothing left, I polish carefully and inject into the pockets a few drops of pure lactic acid two or three times a week until results are produced, which is slow in some cases, but always sure. I then instruct the patient carefully as to the correct way to brush the teeth and gums. To say the least of it, I believe and hope I am effecting cures along this line.—Dr. J. H. Nicholson, *Era.*

**An Effective Ligature.** In cases where the clamp is objectionable for the retention of the rubber dam, and where the ordinary floss is not sufficiently bulky to prevent the rubber from drawing over it, a most admirable method of using the ligature is to first pass the floss through two pieces of rubber tubing, allowing one piece on the buccal and one on the lingual side of the tooth. This is much preferable to stringing beads on the ligature or using other agents as already suggested. The tubing should be the smallest size sold at the rubber stores, the kind used for slipping over the bows of glasses where they rest on the ears. To insure against leakage, drop a little sandarac varnish between the tubing and the enamel on the buccal and lingual sides. E. M. S. Fernandes, in *Review.*

**Practical** If there be one species of cant more detestable than another, it is that which eulogizes what is called the practical man as contradistinguished from the scientific. If by "practical man" is meant one who, having his mind well stored with scientific and general information, has his knowledge chastened and his theoretic temerity subdued by varied experience, nothing can be better; but if, as is commonly meant by the phrase a practical man means one whose knowledge is only derived from habit, or traditional system, such a man has no resource to meet unusual circumstances. Such a man has no plasticity; he kills a man according to rule, and consoles himself like Moliere's doctor, by the reflection that a dead man is only a dead man, but a deviation from received practice is an injury to the whole profession.—Wm. R. Grove, *Cosmos.*

**Cold Water for Resined Impressions.** Some dentists coat plaster impressions with a solution of soap to facilitate the parting of it from the cast; others use a varnish made of one of the resins dissolved in alcohol. To make impression and cast part company readily it is customary to throw both while still in apposition into water, preferably cold except in those cases in which soap or other non-resinous agent has been used as a separating medium. If thrown into hot water the heat, penetrating to the resin coated surface of the impression, effects adhesion, with the result that one has two surfaces cemented each to the other, that part with difficulty and mutually roughen each other, leaving the surface of the cast pitted and scaled in some places and with bits of the impression adhering in others. The safe plan is to soak the pair of mixes for a few minutes in cold water when a resinous parting fluid is used; in hot or cold when the medium is non-resinous. As stated above there is a quicker result when hot water is used, the heat playing an important part in tearing the pieces from each other.—*Office and Laboratory.*

**New Property of Aluminum.** A German investigator has discovered an exceedingly valuable and important property of aluminum, which consists in its application as a whetting agent, the effect produced on cutlery set with it being most astonishing. Though a metal, aluminum possesses the structure of a fine stone, has a strong dissolving power, and developes, upon use for honing, an exceedingly fine metal-setting substance of greasy feel, while showing great adhesion to steel. The knives, etc., treated with it quickly obtain such a fine razor-like edge that even the best whetstone cannot produce a like result. Thus, knives which had been carefully set on a whetstone, when magnified a thousand times still exhibited irregularities and roughness on the edge, while the edge of knives sharpened on aluminum, upon exactly the same magnification, appeared as straight, smooth line.—*Scientific American.*

**Hydrogen Dioxid. Blind Abscesses and Barbed Broaches.** The criticisms that have been published on peroxide of hydrogen amount almost to its condemnation as a practical agent in connection with dentistry.

I wish to enter my protest to these extreme views—it is an exceedingly valuable agent, and I know of nothing that could take its place for certain purposes. All that is needed it seems to me, is a little word of caution, as to its forcible injection into any sac or cavity with insufficient vent. For example, if there is doubt as to the connection between the root canal of a tooth and an apparent sinus or fistula, the fact of such connection should be established by the injection first of simple warm water, or water containing but a small percent of the $H_2 O_2$. A very small amount of the oxygen set free will show demonstrative bubbles sufficiently to establish the diagnosis. When the fact of a free channel has been established the ordinary peroxide can be used with impunity and any small syringe, and without possibility of the calamatous consequences we have had depicted.

As to the treatment of the "blind" abscess I should like to say amen to what was said by Dr. Arthur Black, as to the surgical necessity in many cases of making an opening through the alveolar wall to the end of the root. One might as well try to clean a room by sticking a broom through the transom of a door, as to cure the cases he describes by medication through the hair-sized and often indeterminate root canal.

Dr. Johnson's statement that he had not put a barbed broach in the canal of a tooth for ten or fifteen years surprised me. I wish he had told us what he uses as a substitute. I have tried every sort, I believe, that was ever invented—hook, twist and screw broaches, and the combination of gimlet and fine barbs. And I have tried all makes of barbed broaches, and at last gone back to the Donaldson barbed and smooth, for every day and all the time dependableness.—Garrett Newkirk, in *Review*.

**Making the Cap and Pin (Base of the Porcelain Crown) Easily Removable.**

Having taken the impression and bite with the modeling compound in the usual manner, to show the relation of the occluding teeth, with a small spatula fill the cap smoothly and evenly with either plaster or modeling compound. Soap or oil the surface of this together with the whole impression—both sides. This leaves the pin protruding for the guide. Now take a piece of German silver hollow joint wire (such as may be obtained at any jeweler's supply house, in various sizes), one that just nicely fits over the pin, and of the same length, slip it over the pin for the impression; pour both sides of the impression attaching to the articulator in the usual manner. When plaster is thoroughly set, remove the modeling compound under dry heat. The base of the crown is then at once separated from the model, the tube insuring accuracy of position in building up the crown. I would like here to emphasize one or two points in connection with use of modeling compound. First, the necessity of thorough chilling with cold water before removal from the mouth.

Second, the use of dry heat instead of hot water always for removal from an investment. Garrett Newkirk, in *Review*.

**To Prevent Alloying of Crowns by Fusible Metal.** First, never use it. It so happens, however, that many prefer this method of shaping shells, and to get rid of the metal throw shell and its low-melting contents into a basin of boiling water, the basin still over the flame; or worse, they heat the shell in the flame itself. In the first instance the shell is in contact with the bottom of the basin, and for the time being becomes a part of that utensil; as a consequence it drinks in a small quantity of the fusible metal die, and notwithstanding its treatment later to a bath in acid it is alloyed to some degree and for all time. In the second instance, no matter how skillful the workman, some "dross" clings to the inner surface of the crown; its perfect removal is next to impossible. The effect of this maltreatment shows in the melting down of the alloyed part during annealing or re-inforcing with solder; or, if it escape this fatality, in the "greening" of the crown.

**A Wasteful Lot.** Dentists are ever ready to complain of the non-remunerative character of their work, charging it up to want of appreciation on the part of the public, the public's inability to place a fair value on the service rendered, and what not. These with other not easily controlled causes carry more or less weight in accounting for the chronic impecuniousness of the vast majority of the profession. There are other causes, however, and not the least is our proverbial wastefulness. We have seen dentists, whose financial status was such that they couldn't afford to waste a nickle, throw small clippings of gold on the floor, scatter fillings of the same metal about the bench and chair, sweep the grindings of a crown or bridge from the lathe table and wonder withal why they had failed utterly to get a dollar to the good.

A jeweler in the operating chair happened to see a scrap of foil on the floor, and asked—naturally for him, for he works in gold—"What do you do with your sweeps, Doctor?" He got for an answer: "Oh, they don't amount to anything; thrown into the rubbish can, I guess." "That may be well enough for you, but if I couldn't get a suit of clothing a year out of what you seem to waste I'd be greatly disappointed. You men have no appreciation of the value of gold, except perhaps when you buy it."

At a meeting of a near-by local society Dr. F. M. Rood, in a side discussion on this very subject, said he had recently sent to a refiner four ounces of polishing strips and disks for which he received two dollars and forty cents; had he offered the strips to anyone of the majority of those present at ten cents a pound it is a question if the offer would have been accepted. And yet the poor men of the profession are throwing away annually strips, disks, fillings, clippings, grindings, etc., by the thousands of pounds, a suggestion of which, if done by an employee in a manufacturing jeweler's establishment, would lead to manslaughter in the first degree. And yet we go on wondering why we have to wear last year's hat, shoes run down in the heel, and trousers bewiskered at the ends of the legs.—*Office and Laboratory.*

